# Associations of nutritional knowledge with dietary patterns and breast cancer occurrence

**DOI:** 10.1038/s41598-025-09931-x

**Published:** 2025-07-09

**Authors:** Beata Stasiewicz, Maciej Biernacki, Malgorzata Anna Slowinska, Lidia Wadolowska

**Affiliations:** 1 Department of Human Nutrition, Faculty of Food Science, University of Warmia and Mazury in Olsztyn, Sloneczna 45f, 10-718 Olsztyn, Poland; 2 Department of General and Minimally Invasive Surgery, University of Warmia and Mazury in Olsztyn, 10-045 Olsztyn, Poland

**Keywords:** Breast cancer, Nutritional knowledge, Dietary pattern, Mediterranean diet, Case-control study, Polish women, Cancer, Health care

## Abstract

It is well-established that women’s nutrition knowledge (NK) is strongly associated with children’s nutritional outcomes. However, the association of women’s NK with their own diet and health status, including cancer occurrence, remains unknown. This case-control study aimed to assess the associations of NK with dietary patterns (DPs) and breast cancer (BC) occurrence in peri- and postmenopausal women. Data were collected for 417 women aged 40.0–79.9 years from north-eastern Poland, including 189 BC cases. The Dietary Habits and Nutrition Beliefs Questionnaire (QEB) was used to assess NK. Based on the dietary data collected with a food frequency questionnaire (62-item FFQ-6), the three DPs were previously identified, and the Polish-adapted Mediterranean Diet (Polish-aMED) score was calculated. The odds ratio of BC occurrence was lower at the higher level of NK (16–25 points) by 49% (p = 0.0280) compared to the lower level of NK (0–12 points). A higher level of NK was associated with higher adherence to the ‘Prudent’ pattern and the Polish-aMED score, as well as with lower adherence to the ‘Western’ and the ‘Processed plant fats and sweetened dairy’ patterns compared to the lower level of NK. In conclusion, the high level of nutrition-related knowledge seems to be associated with pro-healthy food choices and cancer prevention. Due to the case-control design of the present study and potential reverse causation, there is a need to confirm the obtained findings in further longitudinal studies.

**Supplementary Information:**

The online version contains supplementary material available at 10.1038/s41598-025-09931-x.

## Introduction

Breast cancer (BC) is one of the greatest health hazards among women^1,2^. It is estimated that over 2 million women are diagnosed with BC each year worldwide, and over 600,000 die from this cause^1^. According to the International Agency for Research on Cancer (IARC), BC accounted for approximately 24% of all female cancer incidence and 15% of all female cancer mortality globally in 2022^1^. Similarly, based on the Polish National Cancer Registry data, it was estimated that BC accounted for 24% of all female cancer cases and 16% of female cancer deaths among Polish women in 2023^2^. The risk of BC increases with age. Around 80% of all BC cases are observed among women over 50^2^. The etiology of BC is complex, and besides age, there are some other established risk factors for BC, including alcohol consumption, sedentary lifestyle, and obesity^3^. Obesity, caused by positive energy balance and characterised by multifactorial aetiology, is associated with cancer risk through several pathways^4^. Excessive energy intake increases the number of free oxygen radicals, leading to DNA damage, thereby inducing carcinogenesis^3,4^. Excessive visceral fat tissue is a source of oestrogen precursors and may increase the risk of oestrogen-receptor-positive breast cancers. Metabolic and hormonal disorders and chronic inflammation associated with obesity may be linked with increased levels of the Insulin-like Growth Factor 1 (IGF-1), and promote the induction of carcinogenesis^3,4^. The impact of diet on the risk of BC is difficult to clearly determine due to the many dietary components and possible interactions between them. Hence, in the assessment of these complex associations, instead of single food items or nutrients, a holistic approach based on dietary patterns (DPs) is taken into account^5^. The recent systematic reviews and meta-analyses have indicated the beneficial impact of DPs composed of vegetables, fruits, legumes, nuts and seeds, fish, and olive oil and described as ‘Prudent’ or based on the Mediterranean diet model in reducing the risk of BC^6,7^. In turn, DPs named ‘Western’ composed of ultra-processed foods with high content of fat and sugar were associated with a higher risk of BC^7^.

**Fig. 1:**
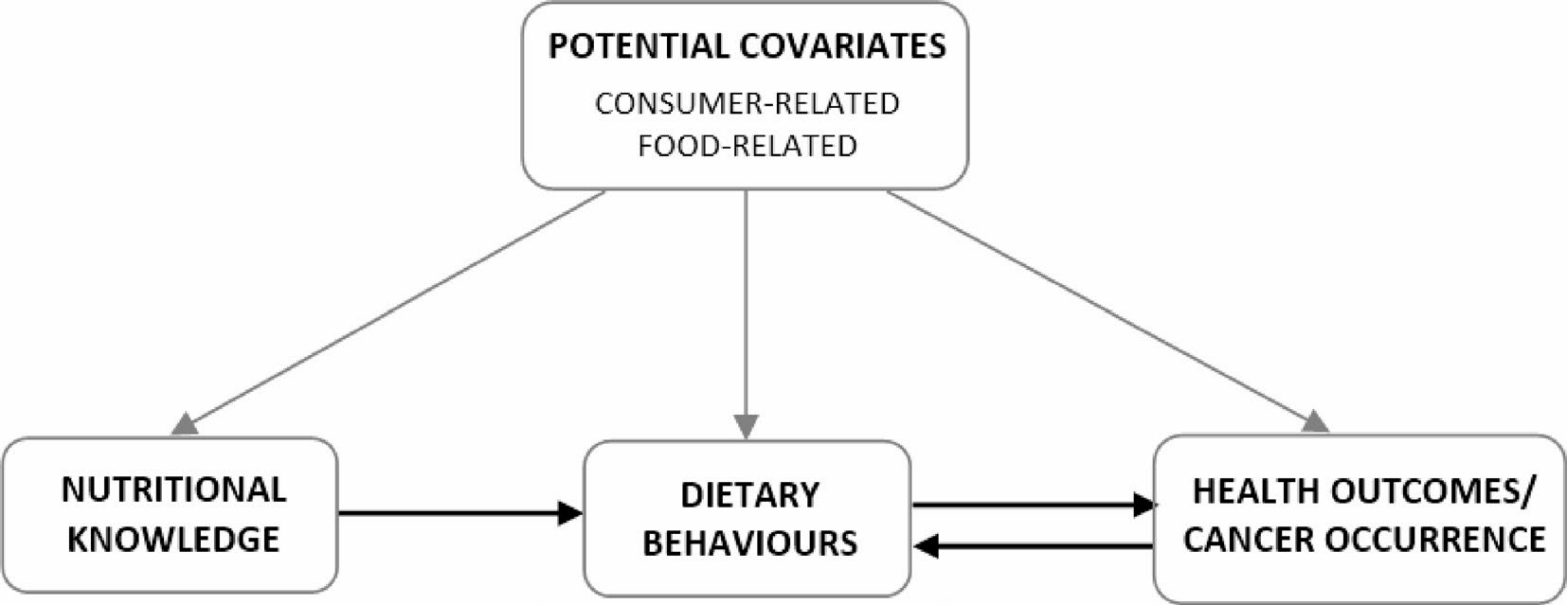
The authors’ concept of the directed acyclic graph (DAG) of the hypothesised links between nutritional knowledge, dietary behaviours, and cancer occurrence.

Knowledge regarding dietary and lifestyle recommendations should reduce the risk of non-communicable diseases, including cancer, through health-promoting behaviour^8^. Nutritional knowledge (NK) may influence dietary intake through more attention to understanding nutrition information on food labels and making healthful decisions about food choices^9^. However, NK is not always applicable in practice. Behavioural science evidence has shown that knowledge is a necessary but insufficient condition for behaviour change^10,11^. According to the health behaviour models, three main kinds of beliefs that determine an individual’s intention to perform a specific behaviour: (1) beliefs about a behaviour, which translate attitudes into behaviours, (2) normative beliefs, which relate to the subjective norms, and others’ attitudes toward a behaviour, and (3) control beliefs based on the perceived ability to perform the behaviour^11^. Regarding dietary behaviours (DB), there are many barriers between knowledge and healthy food choices, including healthy food access, income, and a job^12–17^. Studies revealed that women, especially professionally active ones, often have difficulty finding time to prepare healthy meals and balancing the responsibilities resulting from job and family commitments^17^. The hypothesis of the links between NK, DB and health outcomes (HO), e.g. cancer occurrence was presented on the directed acyclic graph (DAG; Fig. 1). These complex associations involve multiple pathways with many potential covariates, including consumer-related (e.g. demographic, socioeconomic, and lifestyle) and food-related factors (e.g. organoleptic features, packaging and markings, price, and brand)^12–17^. Analysing the links between NK, DB and HO requires careful consideration of causal pathways, assumptions, and the risk of biases, including collider bias^18^. The example of collider bias is the influence of both NK and HO on DB. It may be caused by the selection bias of selecting participants with high NK or cancer survivors who are more aware of the impact of nutrition on health. Due to the reverse causality (e.g., post-diagnosis behaviour change), this may distort the real association between NK and health outcomes^18^. Another important issue involves the sources from which women obtain nutrition information. Acquiring accurate and adequate nutrition information is important as it could inform nutritional choices positively and promote the maintenance of a healthy nutritional status^19^.

In general, women in comparison to men have significantly higher NK, are much more concerned with choosing appropriate food, are more aware of the link between diet and health, and more often implement a healthy, well-balanced diet^20–22^. It is well-established that women’s nutrition knowledge is strongly associated with children’s nutritional outcomes^14,23^. Although many studies carried out on education-based programs have focused on healthy eating among children and adolescents, students, and some specific groups of adults, such as pregnant women or athletes^24–28^there are no studies focused on nutrition-related knowledge and women’s health hazards, including breast cancer occurrence. Available research in this context examines only the level of women’s knowledge about breast cancer screening and self-examination^29–32^ or concerns dietary-lifestyle interventions among BC survivors^33–35^. Hence, this study aimed to assess the associations of nutrition-related knowledge with adherence to dietary patterns and breast cancer occurrence in peri- and postmenopausal women.

## Materials and methods

### Ethical considerations

The study was approved by the Bioethics Committee of the Faculty of Medical Sciences, University of Warmia and Mazury in Olsztyn on 2 October 2013 (resolution no. 29/2013). All subjects gave their written informed consent for inclusion before they participated in the study.

### Study design and sample selection

**Fig. 2:**
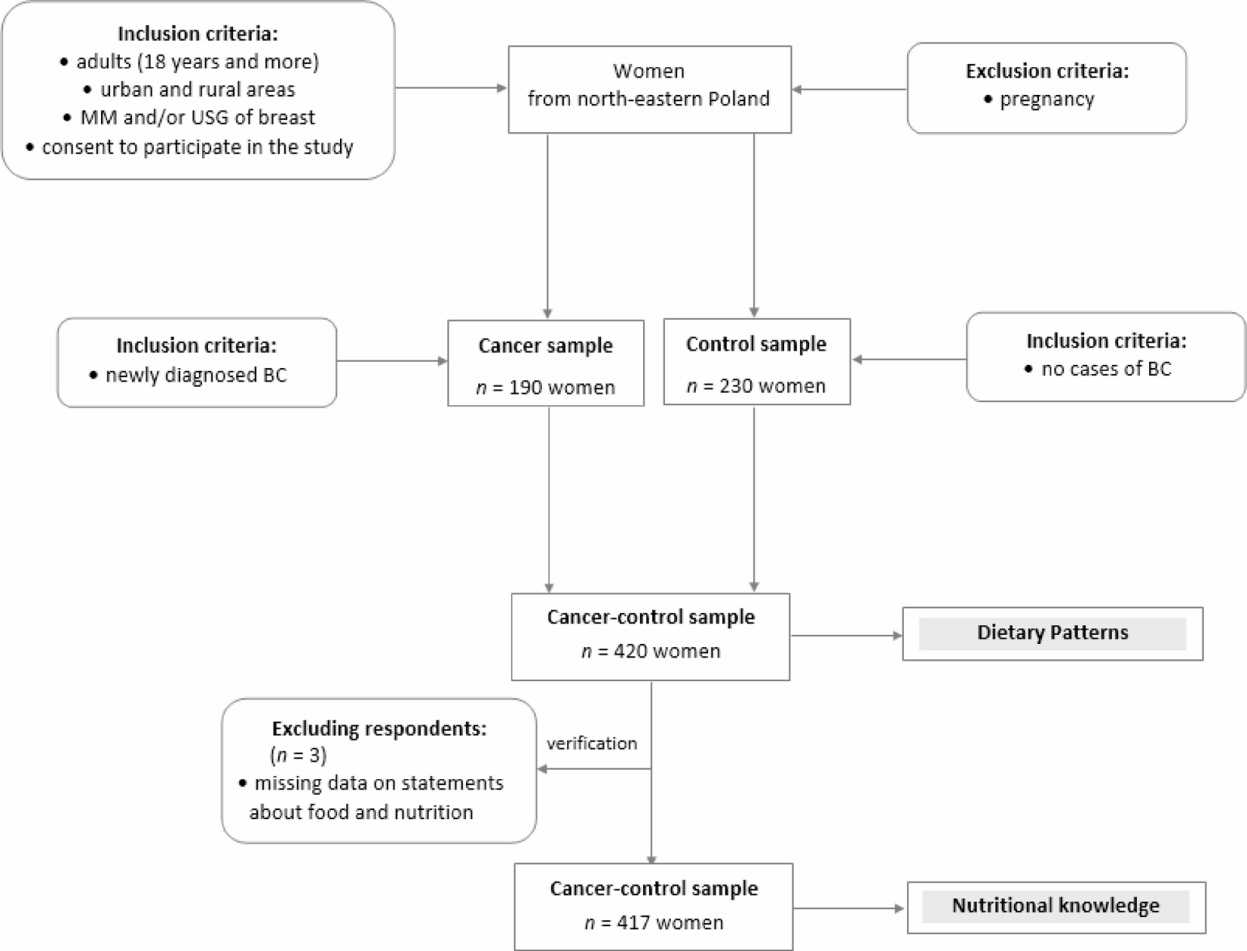
Study design and sample selection. BC—breast cancer; MM—mammography; USG—ultrasonography; the stage of the study is shaded.

This case-control study was conducted in 2014–2017 among women from north-eastern Poland. The cancer-control sample involved 420 subjects, aged 40.0–79.9 (mean 59.9) years, including 190 breast cancer (BC) cases (cancer sample) and 230 women without breast cancer (control sample). All BC cases were newly diagnosed and histologically confirmed at the surgical oncology ward at the Warmia-Masuria Cancer Centre of the Ministry of the Interior and Administration Hospital, Olsztyn, Poland. Details of sample selection, including the molecular–hormone receptor status of the BC subtype, were described previously^36^. Women without any breast pathology based on mammography (MM) and/or breast ultrasonography (USG) within a national breast cancer screening program at medical centres in north-eastern Poland were the control sample. The inclusion and exclusion criteria of sample selection are shown in Fig. 2.

### Assessment of nutritional knowledge and sources of nutrition information

The Dietary Habits and Nutrition Beliefs Questionnaire (KomPAN) in the interviewer-administered and original version known as the Questionnaire of Eating Behaviours (QEB), developed by the Committee of Human Nutrition, Polish Academy of Sciences, and the Manual for Developing Nutritional Data procedure were used to assess nutrition-related knowledge^37^. The reproducibility of KomPAN in dietary habits, lifestyle, and nutrition knowledge assessment among Polish adolescents and adults was reported by Kowalkowska et al.^38^ All subjects were asked the 25 statements concerning food and nutrition knowledge of KomPAN. The set of these statements with the correct answers was included in the Supplementary Table S1. Full data were collected for 417 women, including 189 BC cases and 228 controls. Each time, subjects had to choose one of three answers: “True”, “False”, or “Unsure”. The next step was the recoding of answers. For each correct answer (“True” or “False”), one point was assigned and for each wrong answer (“True” or “False”) or “Unsure”, zero points were assigned. For each subject, all the points were then summarised (ranged 0–25 points). The NK score was evaluated according to the tertiles of total points and interpreted as the following three levels: low (0–12 points), average (13–15 points), and high (16–25 points). The details of the NK score distribution for the total sample are shown in the histogram (Supplementary Figure S1). The created groups of subjects were used in further analyses to compare the dietary patterns and BC occurrence among women with different levels of NK. The correct answers for the set of statements concerning food and nutrition of the KomPAN questionnaire by the case-control status and the level of NK are shown in Supplementary Tables S2 and S3, respectively.

The women were asked about the source(s) from which they obtained information about food and nutrition. There were seven possible sources to choose from: education, healthcare, family, the Internet, radio and television, newspapers, and advertising. In this part, the women could give one or more answers. The responses obtained were used in further analyses among groups with different levels of NK. Sources of nutrition-related information among BC cases and controls were given in Supplementary Table S4.

### Dietary patterns identification

Dietary patterns (DPs) in association with BC occurrence was the main subject of the previous paper, where details of DPs identification were provided^36^. Briefly, dietary data were collected using a validated food frequency questionnaire (62-item FFQ-6)^39^. DPs were determined using the Principal Component Analysis (PCA). The input variables were the frequency of consumption per day of 21 food groups aggregated from 62 food items. The criteria for dietary pattern identification and value of factor loadings for food groups in PCA-derived DPs are given in Table 1. Three DPs were identified. The ‘Western’ DP, previously known as the ‘Non-Healthy‘^36^was characterised by the consumption of refined cereals, red/processed meats, sugar/honey/sweets, potatoes, animal fats, vegetable oils, and sweetened beverages/energy drinks. The ‘Prudent’ DP was characterised by the consumption of fruit, fish, legumes, milk/fermented milk drinks/cheese curd, wholemeal cereals, fruit/vegetable/vegetable-fruit juices, eggs, vegetables, nuts/seeds, vegetable oils, breakfast cereals and cheese. The ‘Processed plant fats and sweetened dairy’ DP, also known as the ‘Margarine and Sweetened Dairy‘^36^was characterised by the consumption of margarine/mayonnaise/dressings, sweetened milk beverages/flavoured cheese curds, white meat, and breakfast cereals. For each DP, scores (in points) and tertile intervals were calculated in further analyses. The mean frequency of food consumption among the case-control sample by tertiles of DPs and the level of nutritional knowledge are presented in Supplementary Tables S5 and S6, respectively.

The Polish-adapted Mediterranean Diet (Polish-aMED) score was described previously^36^. In brief, the Polish-aMED score was calculated based on the frequency consumption (times/day) of seven food items: vegetables, fruit, whole grains, fish, legumes, nuts/seeds, and red/processed meats, and the ratio of the frequency consumption of vegetable oils to animal fat. The values of Pearson’s correlation coefficients for the Polish-aMED score for the case-control sample are shown in Table 1. The criteria for the Polish-aMED score calculation are given in Supplementary Table S7.

**Table 1:** Factor loadings for food groups in PCA-derived dietary patterns and the Pearson’s correlation coefficients for Polish-aMED score among peri- and post-menopausal women (*n* = 420)^36^. Polish-aMED—Polish-adapted Mediterranean Diet (range of points: 0–8); PCA—Principal component analysis; NA—not applied; bolded values are marked for the main components of PCA-derived dietary patterns with absolute loadings ≥ 0.3 and for eight components of the Polish-aMED score; *p < 0.05, test of significance for Pearson’s correlation coefficients. In identifying the number of PCA-derived dps, three criteria were considered: (i) the eigenvalues from the correlation matrix of the standardized variables > 1.0, (ii) the break point identified in the plot of eigenvalues, and (iii) the total variance explained^36^.

Food Groups	PCA-Derived Dietary Patterns			Polish-aMED Score
	‘Western’	‘Prudent’	‘Processed plant fats and sweetened dairy’	
Refined cereals	**0.67**	−0.25	0.12	−0.41 *
Red and processed meats	**0.63**	0.06	0.07	−0.34 *
Sugar, honey and sweets	**0.57**	0.13	0.04	−0.14 *
Potatoes	**0.55**	−0.04	−0.02	−0.20 *
Animal fats	**0.49**	0.12	**−0.66**	−0.31 *
Vegetable oils (including olive oil)	**0.34**	**0.36**	0.02	0.14 *
Sweetened beverages and energy drinks	**0.32**	0.13	0.18	0.01
Fruit	−0.06	**0.55**	−0.05	0.38 *
Fish	−0.07	**0.49**	0.09	0.33 *
Legumes	0.00	**0.48**	0.19	0.36 *
Milk, fermented milk drinks and cheese curd	−0.05	**0.48**	0.13	0.22 *
Wholemeal cereals	**−0.45**	**0.47**	−0.01	0.43 *
Fruit, vegetable, vegetable-fruit juices	0.16	**0.45**	0.00	0.11 *
Eggs	0.20	**0.44**	−0.10	0.10 *
Vegetables	0.00	**0.42**	−0.17	0.34 *
Nuts and seeds	**−0.39**	**0.42**	−0.12	0.46 *
Breakfast cereals	0.04	**0.35**	**0.31**	0.14 *
Cheese	0.23	**0.34**	0.10	0.02
Other fats (margarine, mayonnaise, dressings)	0.17	−0.12	**0.80**	−0.05
Sweetened milk beverages and flavoured cheese	0.29	0.28	**0.36**	−0.06
White meat	0.22	0.08	**0.31**	−0.03
Ratio of vegetable oils to animal fat	NA	NA	NA	0.38 *
Share in explaining the variance (%)	13	12	7	NA

### Statistical analysis

The continuous variables (e.g. age, BMI, socioeconomic status index, NK score expressed in points, DP scores expressed in points) were presented as means and standard deviations (SDs)^40^. These and other socioeconomic, lifestyle, reproductive, and medical variables, including BC cases, were also categorised (e.g. NK score expressed in tertiles (levels), sources of nutrition-related information, DPs expressed in tertiles or levels). The categorical variables were shown in sample percentages. The differences between NK level groups were verified with a non-parametric Kruskal-Wallis test (continuous variables) or the Pearson Chi^2^ test with Yates’ correction as necessary (categorical variables)^40^.

A logistic regression analysis was performed to estimate the odds ratio (OR) and 95% confidence interval (95% CI) of the adherence to the DPs, including the Polish-aMED score, as well as to estimate the ORs of BC occurrence in association with the level of NK^40^. The reference categories (OR = 1.00) were the low level of NK, the bottom tertiles of each DP, and the control sample. The ORs of the adherence to the DPs and BC occurrence for a one-point increase in NK score were also calculated^40^. Two models were created: the crude model and the model adjusted for the potential confounders. In the ORs of the adherence to the DPs, the set of confounders included age, BC cases, BMI, socioeconomic status (SES), and chronic disorders^3,41^. In the ORs of the BC occurrence assessment, the set of confounders included age, BMI, SES, overall physical activity, smoking status, abuse of alcohol, age at menarche, number of full-term pregnancies, oral contraceptive use, hormone-replacement therapy use, family history of breast cancer in first- or second-degree relative, chronic disorders, vitamin/mineral supplements use, molecular of breast cancer subtypes and dietary patterns^3,7^. A description of confounders is given in Supplementary Table S8. The level of significance of OR was verified with Wald’s test^40^. Statistical analyses were performed using the STATIS-TICA software (version 13.0 PL; StatSoft Inc., Tulsa, USA; StatSoft, Krakow, Poland). The level of statistical significance was considered at a p-value < 0.05^40^.

## Results

### Baseline sample characteristics

The baseline characteristics of the sample by the level of NK are shown in Table 2. The percentage of BC cases significantly decreased with the level of NK, and for low, average, and high NK it was 58.5, 45.4, and 31.3%, respectively (*p* < 0.0001). More women with higher NK compared to women with lower NK had high socioeconomic status (36.6 vs. 14.8%; *p* < 0.0001), came from a city (47.0 vs. 31.7%; *p* = 0.0214), had a higher education level (42.5 vs. 16.9%; *p* < 0.0001), better economic situation (19.4 vs. 9.9%; *p* = 0.0055), and a good situation of household (35.1 vs. 19.7%; *p* = 0.0002), were more physically active in leisure time (20.9 vs. 8.5%; *p* = 0.0003), and more frequently used the hormone-replacement therapy (25.4 vs. 12.0%; *p* = 0.0052; Table 2). No difference in the level of NK depending on age, BMI, other lifestyle, reproductive or medical factors was found.

**Table 2:** Sample characteristics by the level of nutritional knowledge (sample percentage or mean (SD). BMI was calculated using measured weight and height; socioeconomic status was calculated on the basis of place of residence, educational level and declared economic situation (description in the material and methods section); physical activity at work: “low” – more than 70% of working time spent sedentary or retired, “moderate” – approximately 50% of working time spent sedentary and 50% of working time spent in an active manner, “high” – approximately 70% of working time spent in an active manner or physical work related to great exertion^37^; physical activity in leisure time: “low” – sedentary for most of the time, watching TV, reading books, walking 1–2 h per week, “moderate” – walking, bike riding, gymnastics, gardening, light physical activity performed 2–3 h per week, “high” – bike riding, jogging, gardening, sport activities involving physical exertion performed more than 3 h weekly^37^; overall physical activity was expressed after combining data based on declared physical activity at work and physical activity in leisure time^36^; abuse of alcohol means at least of 1 bottle (0.5 L) of beer or 2 glasses of wine (300 ml) or 2 drinks (300 ml) or 2 glasses of vodka (60 ml) consumption per day^3^; p-value – level of significance assessed by Chi^2^ test (categorical variables) or Kruskal-Wallis’ test (continuous variables); statistically significant differences between the levels of nutritional knowledge, *p* < 0.05.

Variable	Total sample	Nutritional knowledge level (score in points)			*p*-Value
		Low (0–12)	Average (13–15)	High (16–25)	
Sample Size	417	142	141	134	
Breast cancer cases	45.3	58.5	45.4	31.3	< 0.0001
Age (years)	59.9 (8.6)	60.0 (8.7)	59.6 (9.2)	60.0 (7.7)	0.9285
40.0-49.9	15.6	15.5	19.1	11.9	
50.0-59.9	30.2	31.0	31.2	28.4	
60.0-69.9	42.2	39.4	36.9	50.7	0.2641
70.0-79.9	12.0	14.1	12.8	9.0	
Menopausal status					
Peri-menopausal	14.9	15.5	15.6	13.4	
Post-menopausal	85.1	84.5	84.4	86.6	0.8513
BMI (kg/m^2^)	27.9 (5.0)	28.4 (5.4)	27.9 (4.6)	27.4 (4.8)	0.2965
< 18.5 (underweight)	0.7	0.7	0.0	1.5	
18.5–24.9 (normal weight)	29.4	27.0	27.9	33.6	
25.0-29.9 (overweight)	39.0	39.7	42.1	35.1	0.6186
≥ 30.0 (obesity)	30.8	32.6	30.0	29.9	
Place of residence					
Village	27.8	32.4	32.6	17.9	
Town (< 20 000 inhabitants)	15.3	15.5	17.7	12.7	
Town (20–100 000 inhabitants)	20.6	20.4	19.1	22.4	0.0214
City (> 100 000 inhabitants)	36.2	31.7	30.5	47.0	
Education level					
Primary	13.4	22.5	10.6	6.7	
Secondary	58.3	60.6	63.1	50.7	< 0.0001
Higher	28.3	16.9	26.2	42.5	
Economic situation					
Below the average	16.1	23.2	14.2	10.4	
Average	71.0	66.9	75.9	70.1	0.0055
Above average	12.9	9.9	9.9	19.4	
Situation of household					
We live poorly	0.2	0.7	0.0	0.0	
We live very thriftily	17.0	26.1	13.5	11.2	
We live thriftily	55.6	53.5	63.8	49.3	0.0002
We live well	24.9	19.7	20.6	35.1	
We live very well	2.2	0.0	2.1	4.5	
Socioeconomic status (SES Index)	9.9 (2.1)	9.2 (2.1)	9.7 (1.9)	10.8 (2.0)	< 0.0001
Low	40.8	53.5	45.4	22.4	
Average	36.7	31.7	37.6	41.0	< 0.0001
High	22.5	14.8	17.0	36.6	
Physical activity at work					
Low	53.7	62.0	47.5	51.5	
Moderate	32.9	22.5	37.6	38.8	0.0173
High	13.4	15.5	14.9	9.7	
Physical activity in leisure time					
Low	22.5	28.2	27.7	11.2	
Moderate	64.3	63.4	61.7	67.9	0.0003
High	13.2	8.5	10.6	20.9	
Overall physical activity					
Low	52.5	62.0	51.1	44.0	
Moderate	44.4	36.6	44.0	53.0	0.0228
High	3.1	1.4	5.0	3.0	
Current or former-smokers	53.0	50.7	59.6	48.5	0.1471
Abuse of alcohol	4.1	4.9	4.3	3.0	0.7104
Age at menarche (years)					
< 12	12.2	10.6	12.1	14.2	
12-14.9	63.1	70.4	63.1	55.2	0.1352
≥ 15	24.7	19.0	24.8	30.6	
Number of full-term pregnancies					
0	12.2	10.6	10.6	15.7	
1–2	61.6	58.5	61.7	64.9	0.1912
≥ 3	26.1	31.0	27.7	19.4	
Oral contraceptive use (ever)	20.1	20.4	23.4	16.4	0.3508
Hormone-replacement therapy use (ever)	16.8	12.0	13.5	25.4	0.0052
Family history of BC	19.4	18.3	20.6	19.4	0.9540
Vitamin/mineral supplements use	38.1	31.0	43.3	40.3	0.0857
Chronic disorders	57.3	58.5	55.3	58.2	0.8402

### Sources of nutrition information

**Fig. 3:**
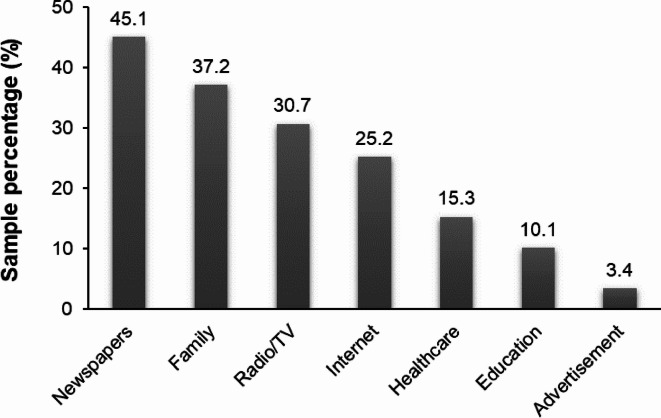
Declared sources of nutrition-related information among women (n=417).Respondents could select one or more sources of nutrition-related information.

Among the analysed sources of nutrition information, most women indicated newspapers (45.1%), family (37.2%), radio and television (30.7%), and the Internet (25.2%; Fig. 3). The fewest women indicated advertisement as a source of nutrition information (3.4%). However, the use of the above-mentioned sources was not significantly related to the level of women’s nutritional knowledge, except for family.

**Fig. 4:**
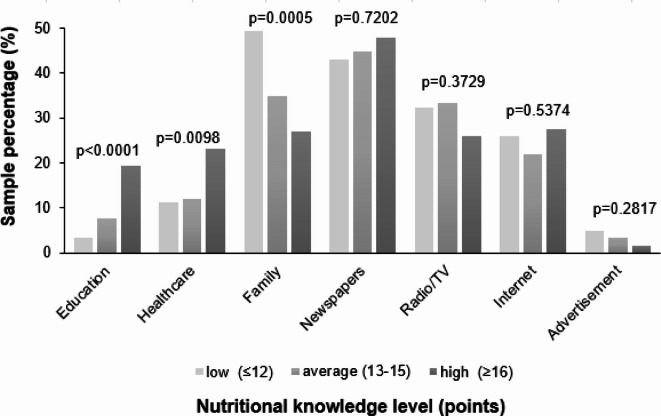
Comparison of the level of nutritional knowledge among women depending on its sources (n=417). Respondents could select one or more sources of nutrition-related information; p-value – level of significance assessed by Chi^2^ test; statistically significant differences between the levels of nutritional knowledge, p<0.05Comparison of the level of nutritional knowledge among women depending on its sources (n=417). Respondents could select one or more sources of nutrition-related information; p-value – level of significance assessed by Chi^2^ test; statistically significant differences between the levels of nutritional knowledge, p<0.05.

The percentage of women who used family advice on nutrition decreased as their level of NK increased (49.3 vs. 34.8 vs. 26.9%; *p* = 0.0005). Conversely, as the level of NK increased, the percentage of women who used healthcare advice on nutrition increased (11.3 vs. 12.1 vs. 23.1%; *p* = 0.0098) or indicated education as a source of nutrition information (3.5 vs. 7.8 vs. 19.4%; *p* < 0.0001; Fig. 4).

### Nutritional knowledge and dietary patterns

**Fig. 5:**
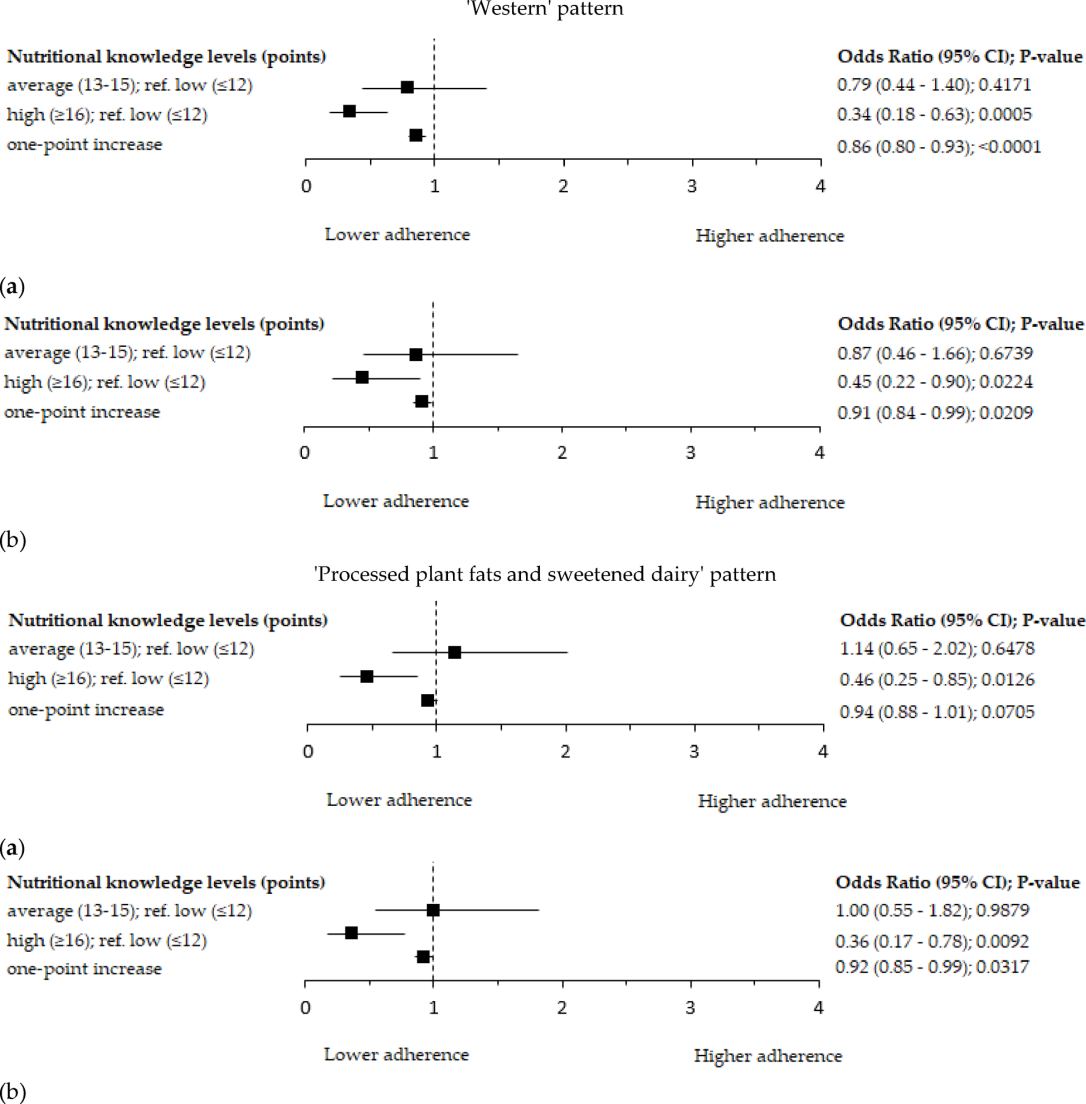
Forest plots of the adherence to the upper tertile of the ‘Western’ and the ‘Processed plant fats and sweetened dairy’ patterns by the level of the nutritional knowledge among peri- and postmenopausal women (n=417): (a) crude model; (b) model adjusted for age (years), breast cancer cases, BMI (kg/m^2^), socioeconomic status (low, average, high), and chronic disorders (no, yes). Ref. - referent, the reference categories were the low level of nutritional knowledge and the bottom tertiles of dietary patterns; 95%CI – 95% confidence interval; p-value – the level of significance was assessed by Wald’s test.

**Fig. 6:**
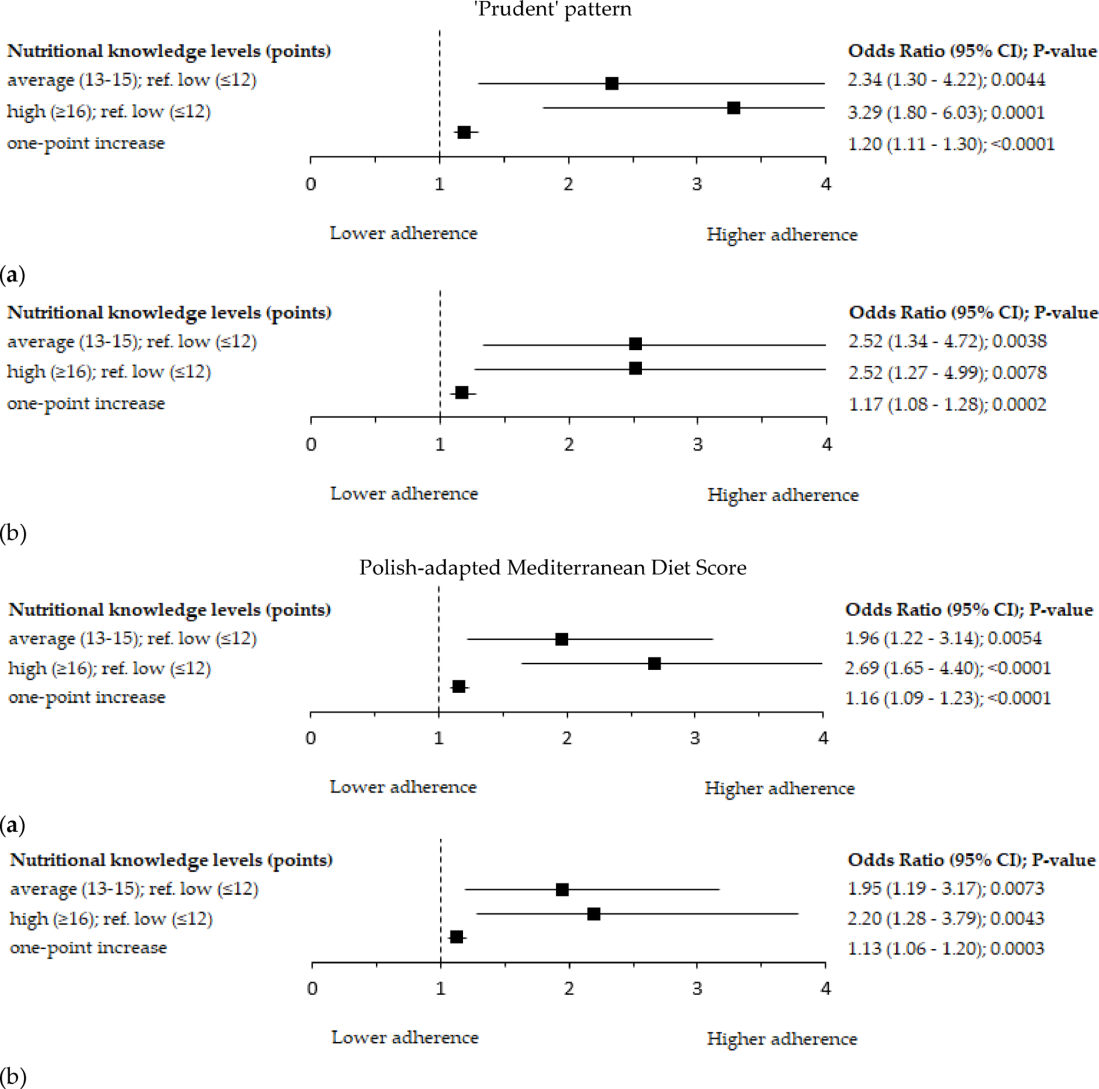
Forest plots of the adherence to the upper tertile of the ‘Prudent’ pattern and the higher level of the Polish-adapted Mediterranean Diet Score (range of points: 0-8) by the level of the nutritional knowledge among peri- and postmenopausal women (n=417): (a) crude model; (b) model adjusted for age (years), breast cancer cases, BMI (kg/m^2^), socioeconomic status (low, average, high), and chronic disorders (no, yes). Ref. - referent, the reference categories were the low level of nutritional knowledge and the bottom tertiles of dietary patterns; 95%CI – 95% confidence interval; p-value – the level of significance was assessed by Wald’s test.

Significant associations between the level of NK and the level of adherence to each of all analysed DPs were observed (Table 3). As the level of NK increased, there was a decrease in the percentage of women in the upper tertiles of the ‘Western’ (40.8 vs. 37.6 vs. 21.6%; p = 0.0067) and the ‘Processed plant fats and sweetened dairy’ pattern (36.6 vs. 41.1 vs. 21.6%; p = 0.0116). Women with higher NK had a lower mean score of the ‘Western’ and ‘Processed plant fats and sweetened dairy’ patterns than women with lower or average levels of NK. On the other hand, as the level of NK increased, there was an increase in the percentage of women in the upper tertile of the ‘Prudent’ pattern (23.9 vs. 36.2 vs. 41.8%; *p* = 0.0015), and in the higher level of adherence to the Polish-aMED score (41.5 vs. 58.2 vs. 65.7%; *p* = 0.0002; Table 3). Women with higher NK had a higher mean score of the ‘Prudent’ and the Polish-aMED patterns than women with lower or average levels of NK. These associations were confirmed in a logistic regression analysis (Figs. 5 and 6). The mean of the consumption of food groups involved in PCA-derived DPs by the level of NK among peri- and post-menopausal women is shown in Supplementary Table S1.

The odds ratio of the ‘Western’ and the ‘Processed plant fats and sweetened dairy’ patterns in the upper tertiles (reference: bottom tertiles) was lower at the higher level of NK by 55% (OR = 0.45; 95% Cl: 0.22–0.90; p = 0.0224) and 64% (OR = 0.36; 95% Cl: 0.17–0.78; p = 0.0092; adjusted models; reference: lower level of NK; Fig. 5), respectively. A one-point increase in the NK score decreased the occurrence of the ‘Processed plant fats and sweetened dairy’ and the ‘Western’ patterns in the upper tertiles by 8% (OR = 0.92; 95% Cl: 0.85–0.99; *p* = 0.0317) and 9% (OR = 0.91; 95% Cl: 0.84–0.99; *p* = 0.0209; adjusted models), respectively.

The odds ratio of the ‘Prudent’ pattern in the upper tertile (reference: bottom tertile) was approximately two and a half times greater at the average (OR = 2.52; 95% Cl: 1.34–4.72; *p* = 0.0038) or higher level of NK (OR = 2.52; 95% Cl: 1.27–4.99; *p* = 0.0078; adjusted models; reference: lower level of NK; Fig. 6). The odds ratio of the Polish-aMED score at the higher level (reference: lower level) was approximately two times greater at the average (OR = 1.95; 95% Cl: 1.19–3.17; *p* = 0.0073) or higher level of NK (OR = 2.20; 95% Cl:1.28–3.79; *p* = 0.0043; adjusted models; reference: lower level of NK; Fig. 6). A one-point increase in the NK score increased the occurrence of the Polish-aMED score at the higher level and the ‘Prudent’ pattern in the upper tertile by 13% (OR = 1.13; 95% Cl: 1.06–1.20; *p* = 0.0003) and 17% (OR = 1.17; 95% Cl: 1.08–1.28; *p* = 0.0002; adjusted models), respectively.

### Nutritional knowledge and breast cancer occurrence

**Fig. 7:**
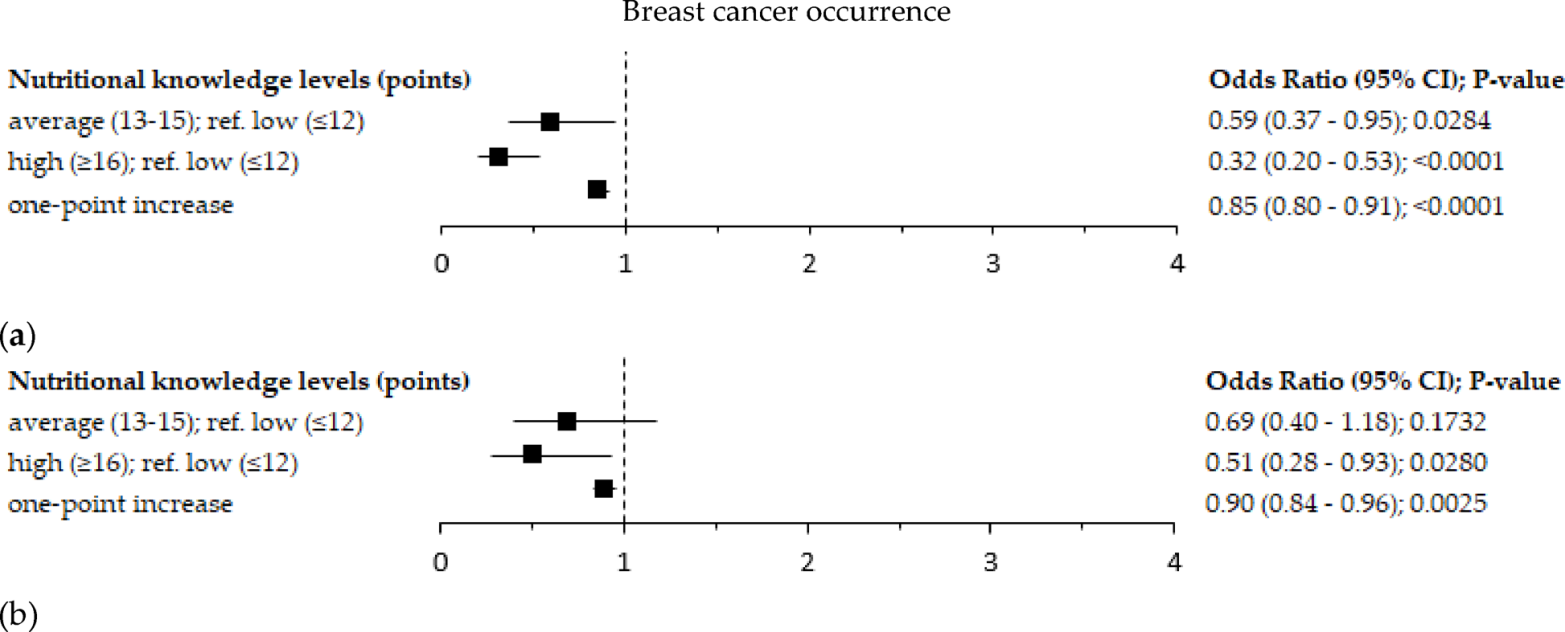
Forest plots of breast cancer occurrence by the level of the nutritional knowledge among peri- and postmenopausal women (n=417): (a) crude model; (b) model adjusted for: age (years), BMI (kg/m^2^), socioeconomic status (low, average, high), overall physical activity (low, moderate, high), smoking status (non-smoker, smoker), abuse of alcohol (no, yes), age at menarche (<12, 12–14.9, ≥15 years), number of full-term pregnancies (0, 1–2, ≥3), oral contraceptive use (no, yes), hormone-replacement therapy use (no, yes), family history of breast cancer in first- or second-degree relative (no, I don’t know, yes), chronic disorders (no, yes), vitamin/mineral supplements use (no, yes), molecular of breast cancer subtypes (triple negative, ER-, PR-, HER2+ subtype, luminal A, luminal B) and the PCA-derived dietary patterns (scores). Ref. - referent, the reference categories were the control sample and the low level of nutritional knowledge; 95%CI – 95% confidence interval; p-value – the level of significance was assessed by Wald’s test.

The odds ratio of breast cancer occurrence was lower at the higher level of NK by 49% (OR = 0.51; 95% Cl: 0.28–0.93; *p* = 0.0280) in the adjusted model and 68% in the crude model (OR = 0.32; 95% Cl: 0.20–0.53; *p* < 0.0001; reference: lower level of NK; Fig. 7). A one-point increase in the NK score decreased the occurrence of breast cancer by 10% in the adjusted model (OR = 0.90; 95% Cl: 0.84–0.96; *p* = 0.0025;) and 15% in the crude model (OR = 0.85; 95% Cl: 0.80–0.91; *p* < 0.0001). The odds ratio of breast cancer occurrence was lower at the average level of NK by 41% (OR = 0.59; 95% Cl: 0.37–0.95; *p* = 0.0284; crude model; reference: lower level of NK). However, this association disappeared after the adjustment for a set of confounders (adjusted model; Fig. 7).

**Table 3:** The level of the nutritional knowledge and dietary patterns (sample percentage or mean (SD). Polish-aMED – Polish-adapted Mediterranean Diet (range of points: 0–8); p-value – level of significance assessed by Chi^2^ test (categorical variables) or Kruskal-Wallis’ test (continuous variables); statistically significant differences between the levels of nutritional knowledge, p < 0.05.

Variable	Total sample	Nutritional knowledge level (score in points)			*p*-Value
		Low (0–12)	Average (13–15)	High (16–25)	
Sample Size	417	142	141	134	
Nutritional knowledge (points)	13.9 (3.5)	10.1 (1.8)	14.0 (0.8)	17.8 (1.8)	< 0.0001
Dietary patterns					
‘Western’ score (points)	3.5 (1.8)	3.9 (1.7)	3.7 (2.0)	3.0 (1.8)	0.0004
Tertiles					
Bottom	33.1	26.8	31.2	41.8	
Middle	33.3	32.4	31.2	36.6	0.0067
Upper	33.6	40.8	37.6	21.6	
‘Prudent’ score (points)	3.4 (1.2)	3.1 (1.3)	3.5 (1.2)	3.6 (1.2)	0.0006
Tertiles					
Bottom	32.9	45.1	29.1	23.9	
Middle	33.3	31.0	34.8	34.3	0.0015
Upper	33.8	23.9	36.2	41.8	
‘Processed plant fats and sweetened dairy’ score (points)	0.1 (1.0)	0.2 (1.1)	0.2 (1.0)	0.1 (0.9)	0.0124
Tertiles					
Bottom	33.6	31.0	30.5	39.6	
Middle	33.1	32.4	28.4	38.8	0.0116
Upper	33.3	36.6	41.1	21.6	
Polish-aMED score (points)	4.7 (1.8)	4.2 (1.7)	4.8 (1.8)	5.0 (1.7)	0.0002
Lower (0–4 points)	45.1	58.5	41.8	34.3	
Higher (5–8 points)	54.9	41.5	58.2	65.7	0.0002

## Discussion

To the authors’ best knowledge, this was the first study that evaluated the associations of nutritional knowledge with dietary patterns and breast cancer occurrence. The obtained findings revealed an inverse association between nutritional knowledge and breast cancer occurrence in peri- and postmenopausal women. As regards dietary patterns, a high level of nutritional knowledge was associated with a high adherence to the ‘Prudent’ pattern and the Polish-aMED score and low adherence to the ‘Western’ and the ‘Processed plant fats and sweetened dairy’ patterns. Therefore, nutrition-related knowledge can successfully translate into practical dietary behaviours and be an important element of cancer prevention. Nevertheless, high NK alone is not sufficient to change eating behaviour^10,11^. Many factors mediate the association between NK and dietary behaviour, including intention, self-efficacy, attitudes and beliefs, as well as perceived behavioural control^10,11^. For example, knowledge that vegetables and fruits are healthy is not sufficient to eat them if there is no intention to buy them. Next, high NK will not be enough to change the diet if self-efficacy is low. Barriers to long-term adherence to dietary recommendations may include growing fatigue, a decline in motivation, and a lack of time to prepare meals^11^. Therefore, to confirm the obtained results and fully explain the connection between NK and dietary behaviour, behavioural constructs that mediate or moderate this association should be taken into account in further studies.

The mean score in nutritional knowledge achieved by women in this study was 13.9 out of 25 points, and was similar to the result obtained by Polish female medical students using the same questionnaire (12.9 out of 25 points)^42^. Close to a 50% threshold of correct answers about nutrition was also noted among male and female adults and young adults came from other European countries, North America, the Middle East, and Africa^19,26,28,43,44^. For example, Maceinaitė et al.^43^ revealed the gaps in knowledge of recommendations for the consumption of various food groups, including the need to increase the consumption of fruit and vegetables and limit the consumption of salt and sweets in research among Lithuanian adults. Lower nutritional knowledge than in the presented study was found among Iranian women, where 10 points were obtained out of 25 points using the KomPAN questionnaire^45^ as well as in the China Health and Nutrition Survey, where only 34.3% of the participants were assessed as having adequate diet-related knowledge^46^.

The present study showed that a higher level compared to the lower level of NK score (16–25 vs. 0–15 points) was associated with a lower occurrence of breast cancer by 49% in pre- and postmenopausal women. This result is difficult to compare with literature data due to the limited number of studies in this area. The only available data comes from the pooled analysis of the authors’ two preliminary case-control studies involving cases of breast cancer in women and lung cancer in men from the Warmia and Mazury region in Poland^47^. This analysis showed that the incidence of both cancers decreased in the upper tertile of NK score (16–25 points) by 94% in comparison with the bottom tertile of NK score (0–15 points)^47^. In the cited study, the same questionnaire was used to assess nutritional knowledge, and the same direction of association was obtained as in the presented paper. However, some limitations related to case-control design and adjustment of the results only to age and type of cancer could have contributed to the overestimation of the findings obtained by Hawrysz et al.^47^. Hence, the careful interpretation of these results is needed. A similar association between nutritional knowledge and healthy status was also obtained in studies focusing on other diseases. For example, the nutritional knowledge of women with polycystic ovary syndrome or coeliac disease patients was worse compared with healthy subjects^48,49^. These results may suggest translating knowledge into practice indirectly and mediating nutrition in the relationship with health^50^. Higher NK probably enabled women to understand the benefits of healthy eating and choose healthy foods by knowing how to read food labels or plan meals. However, to translate NK into practice, the NK-dietary behaviour link was probably strengthened by many predictors of healthy dietary choices, e.g. higher motivation and self-efficacy in making dietary changes, or the ability to prepare foods.

The obtained results suggest that women with high NK were less likely to have breast cancer due to their healthy diet. This hypothesis is supported by the results of previous work from the same case-control study, which showed that the ‘Prudent’ DP and Polish-aMED scores were inversely associated with BC occurrence, whereas the ‘Western’ and the ‘Processed plant fats and sweetened dairy’ patterns increased this risk^36^. Considering the associations between NK and diet, it was observed that women with high nutritional knowledge were characterised by high adherence to the pro-healthy DPs known as ‘Prudent’ or expressed by the Polish-aMED score, and inversely associated with unhealthy DPs such as the ‘Western’ and the ‘Processed plant fats and sweetened dairy’ patterns. Similarly, in the Hawrysz et al.^47^ study, a higher level of nutritional knowledge was associated with the higher quality of a pro-healthy diet expressed in the pro-Healthy-Diet-Index-8 (pHDI-8) and a decrease of the non-Healthy-Diet-Index-8 (nHDI-8). A positive correlation of nutrition literacy with dietary practice was also indicated by Tang et al.^51^ in a multicentre study among Chinese breast cancer patients undergoing chemotherapy. A beneficial effect of NK on diet quality was also found among healthy adults and young adults^21,26,52,53^. These studies showed that greater NK regarding planning a diet was significantly related to healthy dietary habits expressed in increased fibre intake and reduced intake of total fat, saturated fatty acids, and cholesterol^21,26,52,53^. Similar to the current study, low NK, especially related to plant foods, as a barrier to achieving a balanced diet was noted by Varela et al.^54^ among Norwegian and French subjects. Contrary to the present study, research involving athletes from Scotland showed that the ‘good’ NK group had significantly higher intakes of meat and dairy products than those with ‘poor’ NK^28^. These results could be due to greater awareness of current sports nutrition guidelines among the group with higher NK and, hence, the choice of sources of high-quality protein. In turn, Kosendiak et al.^42^ did not observe a significant association between the level of NK and dietary quality among Polish female medical students, except for a greater tendency toward calorie restriction. In general, high NK is often associated with perceived control of food choices and the energy value of meals, especially among young women^42^. The lack of a clear link between NK and diet may result from many mediators, including affective and cognitive attitudes and behavioural factors regarding purchase and cooking abilities^28,45,46,55^. This was supported by an Iranian study in which Sasanfar et al.^56^ obtained the beneficial effects of nutrition education related to cancer prevention based on information, attitudes, and practices related to nutrition and health. After the intervention, Iranian women consumed more whole grains, low-fat dairy, and nuts^56^. These findings are consistent with the results of the presented study, where the same food items were involved in the ‘Prudent’ pattern linked with higher NK and lower BC occurrence.

Among the analysed sources of nutrition information, most women indicated mass media, including newspapers (45.1%), radio/TV (30.7%), and the Internet (25.2%). The news websites (41.8%) were the most common sources of NK in a representative cross-sectional survey among adult Poles^57^. Online resources, mainly the Internet, were the most popular source of nutrition information, especially among younger-age adults, where this percentage of respondents ranged from 67.2 to 92.7%^19^^[,58^^[,59^. Informal or media-based sources, such as social media, blogs, and forums, play an increasingly significant role in shaping public health-related topics, including NK^58–60^. From a public health standpoint, these sources can have both positive and negative influences on NK reliability^60^. Media-based sources provide health information in a broader and more widely accessible way, using simpler language compared to academic or clinical scientific sources. These sources can help rapidly disseminate information about disease outbreaks or food security, e.g., food contamination or recall^60^. However, the informal sources of nutrition-related information are usually not peer-reviewed and do not include expert input, which makes them inaccurate and low quality^60^. Providing very general, often exaggerated information and the lack of citations to the literature indicate the low reliability of these sources of information. Moreover, the mass media are susceptible to misinformation^60^. Uncritically following online sources of nutrition information may result in adverse health effects. Misleading nutrition information can lead to unhealthy behaviours, eating disorders, nutrient deficiencies or supplement abuse^60^. However, the use of mass media was not significantly related to the level of women’s NK. In the present study, only formal education and healthcare advice had a beneficial impact on the increase in women’s NK. The significant importance of formal education in improving NK was observed among students^26^. In turn, in a study by Netzer and Elboim-Gabyzon^59^ involving physical therapists, a majority did not indicate studies as a main source of their nutrition-related knowledge and indicated professional scientific journals as one of the primary sources of nutritional information. Even medical students often report not feeling equipped to provide adequate nutrition care^61^. In the current study, only 10.1% and 15.3% of women declared that formal education and healthcare advice, respectively, were important sources of nutrition information. The reason may be potentially limited access to professional support in terms of nutritional advice within primary healthcare^17^. In Poland, public primary health care still does not include the participation of a dietitian, so the visit is often limited to advice on disease risk factors but without professional nutrition care. Regarding education, specialist nutrition-related knowledge is provided at the stage of higher education in medical and related fields, especially dietetics. The next systemic barriers of the translation of nutritional knowledge into behaviour are infrastructural and digital literacy gaps in rural settings, especially in lower economically advanced countries^62,63^. Greater distance from urban centres complicates access to health care and nutrition education. Many rural areas are poorly connected to the nearest primary health care, lacking roads or public transport^62^. Similarly, lack of the Internet and smartphones with mobile applications limit access to information on food and nutrition or online consultations with a specialist, such as a doctor or dietitian^63^. For example, Brown et al.^63^ found that adolescents who used food, nutrition, and health applications had better results on food, nutrition, and total knowledge than peers who did not use these applications. It seems that the most effective way to increase NK would be to provide nutritional information derived from solid, evidence-based sources presented in an accessible and understandable manner. The benefits of professional education interventions and using virtual tools were indicated by Huang et al.^64^ in a study among BC survivors, where an increase in post-intervention scores was observed in mindful eating practice. The multidisciplinary virtual approach may, therefore, be a good solution for educational programs, especially in the current era of mobile application development. Nevertheless, digital literacy and access challenges, particularly among older adults and rural populations, remain significant barriers^62^. The elderly may lack the skills to use dietary apps and other online tools. Limited internet connection or access to electronic devices can exclude rural residents from digital nutrition initiatives^62,63^. Therefore, currently, nutrition interventions should be based on non-digital, community programs, and support for digital training to ensure inclusivity.

The characteristics of this study sample showed that the factors contributing to higher NK were high socioeconomic status (SES) and its components: place of residence (cities), higher education level, better economic situation, and good household situation. The significant importance of SES, especially the level of education for NK was also indicated among women from Poland, Iran, Sri Lanka, and Austrian, Lithuanian, and Chinese adult populations^15,50,56,57,65^. The higher education level allowed them to understand the specific questions of nutrition-related knowledge and food labels^9,43,50,56,57^. Similar to the current study, the China Health and Nutrition Survey provided evidence that higher levels of dietary knowledge score were associated with urban areas and higher income^12,46^. Low family income may be a barrier not only to obtaining nutritional knowledge but also may have a substantial impact on eating attitudes by limiting food purchases^16,20^. Surprisingly, the monthly income of respondents had no significant influence on Lithuanian nutritional knowledge^43^.

The main strength of this study is the complex evaluation of the associations of NK with dietary patterns and BC occurrence. Furthermore, in the assessment of these links, many potential confounders, including socioeconomic, reproduction, lifestyle, clinical variables, and anthropometric parameters, were taken into account. Although it is not possible in any study to take into account all possible potential confounders. Another strength of the study is that all data were obtained through direct interviews with respondents administered by researchers. Moreover, anthropometric data were not declared but collected based on the measurements. There are also some study limitations, including study design. The case-control design does not allow the assessment of the cause-and-effect relationship between NK and BC occurrence, and limits interpretation of causal pathways^40,66^. These associations were assessed at a one-time point in BC cases and controls. However, all data were collected just after the breast cancer diagnosis (newly BC diagnosed cases), before any education about cancer prevention and treatment. Dietary patterns were identified based on the data of the food frequency consumption within the last 12 months, collected using a retrospective method. This approach could avoid potential reverse causality and prevent false-positive results of the influence of BC diagnosis on dietary patterns or NK^40,66^. Nevertheless, the association of NK – DPs – BC presumes a temporal sequence, which cannot be confirmed in the present study due to its retrospective design. Second, there are other methodological limitations, such as reliance on self-reported dietary data and regional sampling bias. A non-random sample selection may reduce the external validity of the study^66^. For these reasons, the results obtained should be interpreted with caution and not be generalised at the population level. The next limitation is that the women’s NK was assessed based on the limited 25 statements about food and nutrition. Nevertheless, these data were collected using the KomPAN questionnaire, which was originally developed and validated in Poland^38^. This tool was also used by researchers in other countries^42,45^. For example, Cieśla et al. (2022)^67^ and Saadati et al. (2022)^68^ assessed the construct validity of the KomPAN questionnaire among university students from Germany and Slovakia and in the Iranian adult population. These studies showed acceptable to good reproducibility and reliability of the KomPAN questionnaire. This indicates opportunities to use this assessment tool in analyses of the dietary habits and nutritional knowledge not only among Poles. Nevertheless, adaptation, including cultural, translation, and re-validation, is essential to ensure the tool’s generalizability and effectiveness in new regions^66^. Otherwise, using KomPAN outside Poland without local validation may affect the accuracy, relevance, and interpretability of results. The list of food items of KomPAN is specific to Polish cuisine and culture and may not reflect the typical diet elsewhere. Even if the questionnaire is translated, subtle meanings may not be preserved, leading to misclassification^66^. The measurement construct of KomPAN, e.g., dietary quality scores, may also not have the same meaning or implications outside Poland^38^. Lastly, due to the limited BC cases, the total sample size was relatively small, although sufficient to estimate NK with proper statistical power, and was adequate to perform logistic regression analysis^69^.

In conclusion, the results showed that women with greater nutritional knowledge follow whole and minimally processed food-based dietary patterns, including the Polish-adapted Mediterranean Diet, limit adherence to refined and processed food-based patterns, and have a lower odds of breast cancer occurrence. Therefore, a high level of nutrition-related knowledge seems to be associated with making pro-healthy food choices and may contribute to breast cancer prevention in women. However, due to the case-control design and limited sample to the Warmia and Mazury region in Poland, there is a need to confirm these findings in further longitudinal studies involving large samples representative of the women’s population of different countries.

The analysis of sources of information on food and nutrition indicates the need to take many actions to raise the level of nutrition-related knowledge, including nutritional education as part of formal education and primary health care. Special care regarding nutritional education should be given to women who have low socioeconomic status, come from a village, have primary education, and have a worse economic situation, because, in this group, low nutritional knowledge was observed. In the future, an evidence-based intervention plan needs to be developed that focuses on removing barriers to achieving nutritional knowledge and decreasing the prevalence of non-communicable diseases by practising healthy dietary habits.

## Electronic supplementary material

Below is the link to the electronic supplementary material.

Supplementary Material 1

## Data Availability

Data availability of statementThe datasets analysed during the current study are available from the corresponding author upon reasonable request.
