# Supplementary material for: Associations of nutritional knowledge with dietary patterns and breast cancer occurrence
Source: Sci Rep. 2025 Jul 9;15:24656. doi: 10.1038/s41598-025-09931-x (PMC12241588; doi:10.1038/s41598-025-09931-x)
Supplement: Supplementary file 1 — Supplementary Material 1 [file 41598_2025_9931_MOESM1_ESM.doc]

**Table S1.** Correct answers for the set of statements concerning food and nutrition knowledge of Dietary Habits and Nutrition Beliefs Questionnaire (KomPAN), developed by the Committee of Human Nutrition, Polish Academy of Sciences (number of statements in the questionnaire: 55-79)

| Statement | Correct answer | Points |
| --- | --- | --- |
| 55. It is enough to eat wholegrains/cereals once a day. | False | 1 |
| 56. Only children and adolescents should drink milk. | False | 1 |
| 57. Fruit and/or vegetables should be consumed with every meal. | True | 1 |
| 58. Consumption of mouldy bread can result in food poisoning caused by *Salmonella.* | False | 1 |
| 59. High intakes of salt protect from hypertension. | False | 1 |
| 60. Limiting high-fat foods in everyday diet is protective against cardiovascular diseases. | True | 1 |
| 61. Frequent consumption of oily fish contributes to atherosclerosis. | False | 1 |
| 62. Frequent consumption of grilled meats contributes to the onset of cancer. | True | 1 |
| 63. Vegetarian diet increases the risk of anaemia. | True | 1 |
| 64. Bio-yoghurts contain beneficial gut bacteria. | True | 1 |
| 65. Vegetable oils and olive oil contain a high amount of cholesterol. | False | 1 |
| 66. Wholemeal bread have more fibre than white bread. | True | 1 |
| 67. Fruit and vegetables are a source of ‘empty calories’. | False | 1 |
| 68. Butter and fortified margarines have high content of vitamin A and D. | True | 1 |
| 69. Cheese is a better source of calcium than cottage cheese. | True | 1 |
| 70. Offal has high amounts of ‘bad’ cholesterol - LDL. | False | 1 |
| 71. In a healthy diet, complex carbohydrates should be replaced with simple sugars. | False | 1 |
| 72. In a balanced diet, proteins should be the main source of energy. | False | 1 |
| 73. Inadequate intakes of vitamin PP can cause skin inflammation and diarrhoea. | True | 1 |
| 74. Sun exposure increases the synthesis of vitamin D in the human body. | True | 1 |
| 75. Phosphorus is a component of neural tissue. | True | 1 |
| 76. The ratio of calcium to phosphorus in a healthy diet should be 1:1. | True | 1 |
| 77. Consumption of fruit with high content of vitamin C increases bioavailability of iron. | True | 1 |
| 78. Starting cooking vegetables in cold water helps to preserve the nutrients. | False | 1 |
| 79. Sweets and animal fats are particularly high nutrient dense foods. | False | 1 |

| **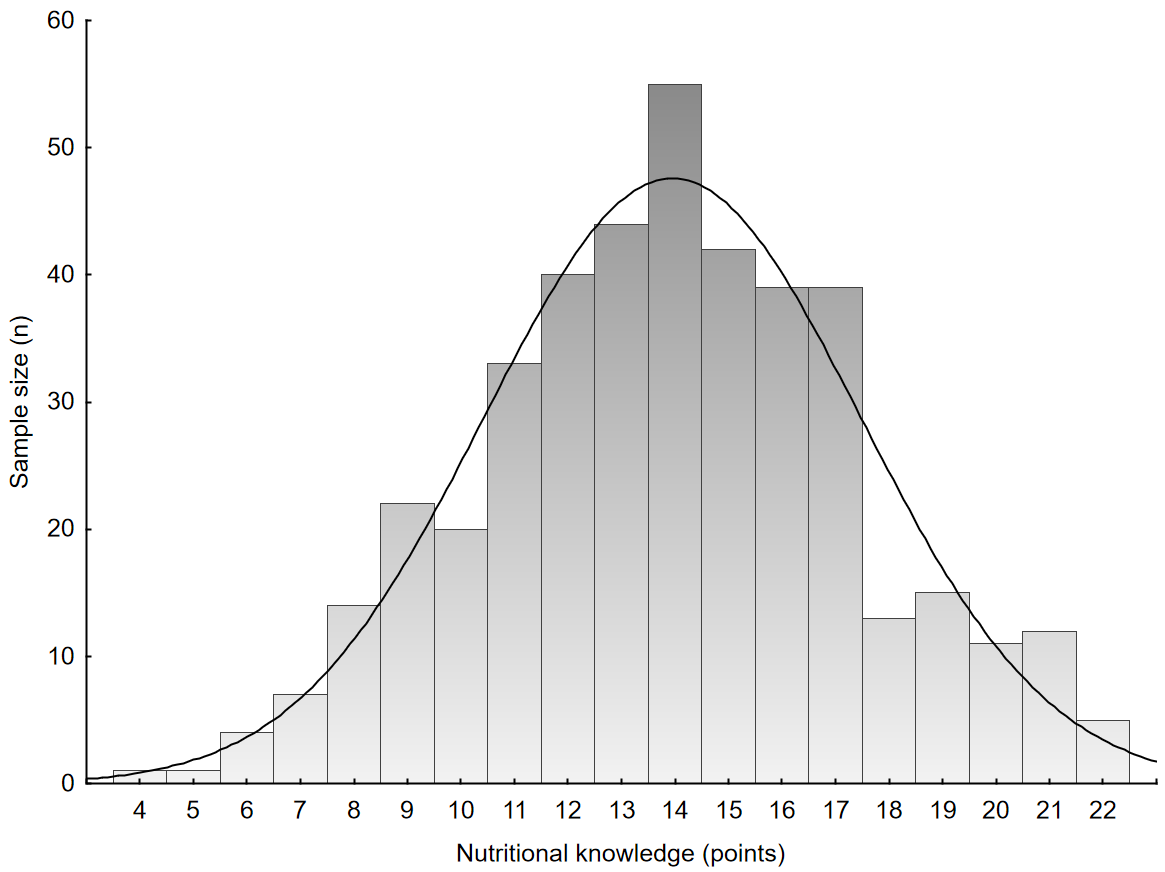** |
| --- |

**Figure S1.** The histogram of the nutritional knowledge distribution (ranged 0-25 points) among peri- and post-menopausal women (n=417)

**Table S2.** Correct answers for the set of statements concerning food and nutrition of the KomPAN questionnaire by the case-control status (number of statements in the questionnaire: 55-79)

| **Statement** | **Correct answers (%)** | | | |
| --- | --- | --- | --- | --- |
|  | **Cancer-Control Sample** | **Cancer Sample** | **Control Sample** | ***p*-Value** |
| Sample Size (n) | 447 | 189 | 228 |  |
| 55. It is enough to eat wholegrains/cereals once a day. | 36.9 | 41.8 | 32.9 | 0.0607 |
| 56. Only children and adolescents should drink milk. | 75.3 | 73.5 | 76.8 | 0.4494 |
| 57. Fruit and/or vegetables should be consumed with every meal. | 72.2 | 68.8 | 75.0 | 0.1584 |
| 58. Consumption of mouldy bread can result in food poisoning caused by *Salmonella.* | 31.7 | 22.2 | 39.5 | 0.0002 |
| 59. High intakes of salt protect from hypertension. | 84.2 | 79.4 | 88.2 | 0.0143 |
| 60. Limiting high-fat foods in everyday diet is protective against cardiovascular diseases. | 88.0 | 83.6 | 91.7 | 0.0116 |
| 61. Frequent consumption of oily fish contributes to atherosclerosis. | 60.4 | 54.5 | 65.4 | 0.0241 |
| 62. Frequent consumption of grilled meats contributes to the onset of cancer. | 66.4 | 64.0 | 68.4 | 0.3436 |
| 63. Vegetarian diet increases the risk of anaemia. | 56.4 | 51.3 | 60.5 | 0.0592 |
| 64. Bio-yoghurts contain beneficial gut bacteria. | 84.7 | 76.2 | 91.7 | <0.0001 |
| 65. Vegetable oils and olive oil contain a high amount of cholesterol. | 61.6 | 54.0 | 68.0 | 0.0034 |
| 66. Wholemeal bread have more fibre than white bread. | 87.3 | 83.1 | 90.8 | 0.0185 |
| 67. Fruit and vegetables are a source of ‘empty calories’. | 78.7 | 75.1 | 81.6 | 0.1097 |
| 68. Butter and fortified margarines have high content of vitamin A and D. | 57.6 | 49.2 | 64.5 | 0.0017 |
| 69. Cheese is a better source of calcium than cottage cheese. | 37.2 | 34.4 | 39.5 | 0.2851 |
| 70. Offal has high amounts of ‘bad’ cholesterol - LDL. | 14.4 | 12.2 | 16.2 | 0.2398 |
| 71. In a healthy diet, complex carbohydrates should be replaced with simple sugars. | 37.2 | 34.4 | 39.5 | 0.2851 |
| 72. In a balanced diet, proteins should be the main source of energy. | 24.5 | 19.0 | 28.9 | 0.0192 |
| 73. Inadequate intakes of vitamin PP can cause skin inflammation and diarrhoea. | 27.3 | 25.4 | 28.9 | 0.4180 |
| 74. Sun exposure increases the synthesis of vitamin D in the human body. | 89.0 | 86.8 | 90.8 | 0.1924 |
| 75. Phosphorus is a component of neural tissue. | 26.6 | 22.8 | 29.8 | 0.1038 |
| 76. The ratio of calcium to phosphorus in a healthy diet should be 1:1. | 19.2 | 21.7 | 17.1 | 0.2362 |
| 77. Consumption of fruit with high content of vitamin C increases bioavailability of iron. | 60.2 | 56.6 | 63.2 | 0.1741 |
| 78. Starting cooking vegetables in cold water helps to preserve the nutrients. | 54.7 | 47.1 | 61.0 | 0.0046 |
| 79. Sweets and animal fats are particularly high nutrient dense foods. | 58.3 | 52.9 | 62.7 | 0.0432 |

% – sample percentage; p-value – level of significance assessed by chi^2^ test.

**Table S3.** Correct answers for the set of statements concerning food and nutrition of the KomPAN questionnaire by the level of nutritional knowledge (number of statements in the questionnaire: 55-79)

| **Statement** | **Correct answers (%)** | | | |
| --- | --- | --- | --- | --- |
|  | **Nutritional knowledge level (points)** | | | ***p*-Value** |
|  | **low**  **(≤12)** | **average (13-15)** | **high**  **(≥16)** |  |
| Sample Size (n) | 142 | 141 | 134 |  |
| 55. It is enough to eat wholegrains/cereals once a day. | 26.1 | 32.6 | 53.0 | <0.0001 |
| 56. Only children and adolescents should drink milk. | 69.0 | 80.9 | 76.1 | 0.0672 |
| 57. Fruit and/or vegetables should be consumed with every meal. | 58.5 | 73.8 | 85.1 | <0.0001 |
| 58. Consumption of mouldy bread can result in food poisoning caused by *Salmonella.* | 13.4 | 29.1 | 53.7 | <0.0001 |
| 59. High intakes of salt protect from hypertension. | 71.1 | 87.9 | 94.0 | <0.0001 |
| 60. Limiting high-fat foods in everyday diet is protective against cardiovascular diseases. | 80.3 | 89.4 | 94.8 | 0.0009 |
| 61. Frequent consumption of oily fish contributes to atherosclerosis. | 40.1 | 63.1 | 79.1 | <0.0001 |
| 62. Frequent consumption of grilled meats contributes to the onset of cancer. | 44.4 | 75.2 | 80.6 | <0.0001 |
| 63. Vegetarian diet increases the risk of anaemia. | 37.3 | 53.2 | 79.9 | <0.0001 |
| 64. Bio-yoghurts contain beneficial gut bacteria. | 69.7 | 88.7 | 96.3 | <0.0001 |
| 65. Vegetable oils and olive oil contain a high amount of cholesterol. | 43.0 | 63.1 | 79.9 | <0.0001 |
| 66. Wholemeal bread have more fibre than white bread. | 71.8 | 92.2 | 98.5 | <0.0001 |
| 67. Fruit and vegetables are a source of ‘empty calories’. | 62.0 | 83.0 | 91.8 | <0.0001 |
| 68. Butter and fortified margarines have high content of vitamin A and D. | 33.8 | 57.4 | 82.8 | <0.0001 |
| 69. Cheese is a better source of calcium than cottage cheese. | 23.2 | 30.5 | 59.0 | <0.0001 |
| 70. Offal has high amounts of ‘bad’ cholesterol - LDL. | 9.2 | 15.6 | 18.7 | 0.0704 |
| 71. In a healthy diet, complex carbohydrates should be replaced with simple sugars. | 18.3 | 32.6 | 61.9 | <0.0001 |
| 72. In a balanced diet, proteins should be the main source of energy. | 7.0 | 22.0 | 45.5 | <0.0001 |
| 73. Inadequate intakes of vitamin PP can cause skin inflammation and diarrhoea. | 11.3 | 24.8 | 47.0 | <0.0001 |
| 74. Sun exposure increases the synthesis of vitamin D in the human body. | 80.3 | 91.5 | 95.5 | 0.0001 |
| 75. Phosphorus is a component of neural tissue. | 9.9 | 21.3 | 50.0 | <0.0001 |
| 76. The ratio of calcium to phosphorus in a healthy diet should be 1:1. | 9.9 | 19.1 | 29.1 | 0.0003 |
| 77. Consumption of fruit with high content of vitamin C increases bioavailability of iron. | 43.7 | 58.2 | 79.9 | <0.0001 |
| 78. Starting cooking vegetables in cold water helps to preserve the nutrients. | 37.3 | 57.4 | 70.1 | <0.0001 |
| 79. Sweets and animal fats are particularly high nutrient dense foods. | 40.1 | 56.0 | 79.9 | <0.0001 |

% – sample percentage; p-value – level of significance assessed by chi^2^ test.

Table S4. Sources of nutrition-related information among breast cancer and control samples (%)

| **Sources of nutrition-related information** | **Cancer-Control Sample** | **Cancer Sample** | **Control Sample** | ***p*-Value** |
| --- | --- | --- | --- | --- |
| Sample Size (n) | 447 | 189 | 228 |  |
| Education | 10.0 | 3.2 | 15.7 | <0.0001 |
| Healthcare | 15.3 | 12.7 | 17.5 | 0.1778 |
| Family | 37.3 | 45.5 | 30.6 | 0.0017 |
| Internet | 25.1 | 20.6 | 28.8 | 0.0548 |
| Newspapers | 45.0 | 46.0 | 44.1 | 0.6935 |
| Radio/TV | 30.6 | 32.8 | 28.8 | 0.3792 |
| Advertisement | 3.3 | 4.8 | 2.2 | 0.1448 |

% – sample percentage; p-value – level of significance assessed by chi^2^ test; statistically significant differences between the pairs of cancer and control samples were considered at the p<0.05.

**Table S5.** The mean (95% CI) of food consumption by dietary patterns among peri- and post-menopausal women (*n* = 420)

| **Food groups** | **Total** | **Dietary Patterns** | | | | | | | | | | |
| --- | --- | --- | --- | --- | --- | --- | --- | --- | --- | --- | --- | --- |
|  |  | **‘Western’**  **tertiles** | | | **‘Prudent’**  **tertiles** | | | **‘Processed plant fats and sweetened dairy’**  **tertiles** | | | **Polish-aMED**  **levels** | |
|  |  | bottom | middle | upper | bottom | middle | upper | bottom | middle | upper | lower | higher |
| Sample size (n) | 420 | 139 | 141 | 140 | 139 | 140 | 141 | 140 | 139 | 141 | 189 | 231 |
| Frequency of food consumption (times/day)^#^ | | | | | | | | | | | | |
| Sugar, honey and sweets | 1.9  (1.7; 2.0) | 1.2  (1.0; 1.3) | 1.7  (1.5; 1.8) | 2.8  (2.6; 3.0) | 1.7  (1.5; 1.9) | 1.9  (1.7; 2.1) | 2.1  (1.8; 2.3) | 2.0  (1.7; 2.2) | 1.7  (1.5; 1.9) | 2.0  (1.8; 2.2) | 2.0  (1.8, 2.2) | 1.8  (1.6, 1.9) |
| Red and processed meats | 1.4  (1.3; 1.4) | 0.8  (0.7; 0.9) | 1.4  (1.3; 1.5) | 1.9  (1.8; 2.0) | 1.3  (1.2; 1.4) | 1.3  (1.2; 1.5) | 1.5  (1.3; 1.6) | 1.4  (1.3; 1.5) | 1.3  (1.1; 1.4) | 1.4  (1.3; 1.5) | 1.6  (1.5, 1.7) | 1.2  (1.1, 1.3) |
| Animal fats | 1.1  (1.1; 1.2) | 0.7  (0.6; 0.8) | 1.1  (1.0; 1.2) | 1.6  (1.5; 1.8) | 1.0  (0.9; 1.1) | 1.2  (1.1; 1.4) | 1.2  (1.1; 1.4) | 1.9  (1.7; 2.0) | 1.0  (0.9; 1.2) | 0.5  (0.5; 0.6) | 1.4  (1.3, 1.5) | 0.9  (0.8, 1.0) |
| Milk, fermented milk drinks and cheese curd | 1.1  (1.0; 1.1) | 1.2  (1.0; 1.3) | 1.0  (0.9; 1.1) | 1.1  (1.0; 1.2) | 0.7  (0.6; 0.8) | 1.1  (1.0; 1.2) | 1.4  (1.3; 1.6) | 0.9  (0.8; 1.0) | 1.2  (1.1; 1.3) | 1.1  (1.0; 1.2) | 1.0  (0.9, 1.1) | 1.2  (1.1, 1.2) |
| Refined cereals | 0.9  (0.9; 1.0) | 0.4  (0.3; 0.4) | 0.9  (0.8; 1.0) | 1.5  (1.4; 1.6) | 1.2  (1.1; 1.3) | 0.8  (0.7; 0.9) | 0.8  (0.7; 0.9) | 0.9  (0.8; 1.1) | 0.8  (0.7; 0.9) | 1.1  (1.0; 1.2) | 1.2  (1.1, 1.3) | 0.7  (0.6, 0.8) |
| Vegetables | 1.2  (1.2; 1.3) | 1.2  (1.1; 1.3) | 1.2  (1.2; 1.3) | 1.2  (1.1; 1.3) | 1.0  (0.9; 1.1) | 1.2  (1.1; 1.3) | 1.5  (1.4; 1.6) | 1.3  (1.3; 1.4) | 1.2  (1.1; 1.3) | 1.1  (1.0; 1.2) | 1.1  (1.0, 1.1) | 1.4  (1.3, 1.4) |
| Fruit | 1.0  (0.9; 1.0) | 1.0  (0.9; 1.1) | 1.0  (0.9; 1.0) | 0.9  (0.8; 1.0) | 0.7  (0.6; 0.8) | 0.9  (0.9; 1.0) | 1.3  (1.2; 1.3) | 1.0  (0.9; 1.1) | 1.0  (0.9; 1.1) | 1.0  (0.9; 1.0) | 0.8  (0.7, 0.8) | 1.1  (1.1, 1.2) |
| Wholemeal cereals | 0.9  (0.8; 1.0) | 1.3  (1.2; 1.4) | 0.9  (0.7; 1.0) | 0.5  (0.4; 0.6) | 0.5  (0.4; 0.6) | 0.9  (0.8; 1.0) | 1.3  (1.1; 1.4) | 0.9  (0.8; 1.0) | 1.0  (0.8; 1.1) | 0.8  (0.7; 0.9) | 0.6  (0.5, 0.7) | 1.1  (1.0, 1.2) |
| Potatoes | 0.6  (0.6; 0.7) | 0.4  (0.3; 0.4) | 0.7  (0.6; 0.7) | 0.8  (0.8; 0.8) | 0.7  (0.6; 0.7) | 0.6  (0.5; 0.6) | 0.6  (0.6; 0.7) | 0.7  (0.6; 0.7) | 0.6  (0.5; 0.6) | 0.6  (0.6; 0.7) | 0.7  (0.6, 0.7) | 0.6  (0.5, 0.6) |
| Vegetable oils | 0.6  (0.6; 0.6) | 0.5  (0.5; 0.6) | 0.6  (0.6; 0.7) | 0.7  (0.7; 0.8) | 0.5  (0.4; 0.5) | 0.7  (0.6; 0.7) | 0.7  (0.7; 0.8) | 0.6  (0.6; 0.7) | 0.6  (0.5; 0.6) | 0.6  (0.6; 0.7) | 0.6  (0.5, 0.6) | 0.7  (0.6, 0.7) |
| Other fats | 0.5  (0.4; 0.5) | 0.3  (0.2; 0.4) | 0.5  (0.4; 0.6) | 0.6  (0.5; 0.7) | 0.6  (0.5; 0.7) | 0.3  (0.2; 0.4) | 0.5  (0.4; 0.6) | 0.1  (0.1; 0.1) | 0.2  (0.1; 0.2) | 1.1  (1.0; 1.2) | 0.5  (0.4, 0.6) | 0.4  (0.4, 0.5) |
| Cheese | 0.4  (0.3; 0.4) | 0.3  (0.3; 0.4) | 0.4  (0.3; 0.4) | 0.4  (0.4; 0.5) | 0.3  (0.2; 0.3) | 0.3  (0.3; 0.4) | 0.5  (0.4; 0.5) | 0.4  (0.3; 0.4) | 0.3  (0.3; 0.4) | 0.4  (0.4; 0.5) | 0.4  (0.3, 0.4) | 0.4  (0.3, 0.4) |
| White meat | 0.4  (0.4; 0.5) | 0.4  (0.3; 0.4) | 0.5  (0.4; 0.5) | 0.5  (0.5; 0.5) | 0.4  (0.4; 0.5) | 0.4  (0.4; 0.5) | 0.5  (0.4; 0.5) | 0.4  (0.3; 0.4) | 0.5  (0.4; 0.5) | 0.5  (0.5; 0.6) | 0.4  (0.4, 0.5) | 0.4  (0.4, 0.5) |
| Juices | 0.5  (0.4; 0.5) | 0.3  (0.3; 0.4) | 0.5  (0.4; 0.6) | 0.6  (0.5; 0.7) | 0.2  (0.2; 0.3) | 0.4  (0.3; 0.5) | 0.8  (0.7; 0.9) | 0.5  (0.4; 0.6) | 0.5  (0.4; 0.6) | 0.4  (0.4; 0.5) | 0.4  (0.3, 0.4) | 0.6  (0.5, 0.7) |
| Eggs | 0.4  (0.3; 0.4) | 0.3  (0.3; 0.3) | 0.3  (0.3; 0.4) | 0.4  (0.4; 0.5) | 0.2  (0.2; 0.3) | 0.3  (0.3; 0.4) | 0.5  (0.5; 0.6) | 0.4  (0.4; 0.5) | 0.3  (0.3; 0.4) | 0.3  (0.3; 0.4) | 0.3  (0.3, 0.4) | 0.4  (0.3, 0.4) |
| Sweetened milk beverages and flavored cheese curds | 0.3  (0.3; 0.4) | 0.2  (0.1; 0.2) | 0.3  (0.2; 0.4) | 0.5  (0.4; 0.6) | 0.2  (0.1; 0.2) | 0.3  (0.2; 0.4) | 0.5  (0.4; 0.6) | 0.2  (0.1; 0.2) | 0.3  (0.2; 0.4) | 0.5  (0.4; 0.6) | 0.4  (0.3, 0.4) | 0.3  (0.2, 0.3) |

| **Table S5.** *Cont.* | | | | | | | | | | | | |
| --- | --- | --- | --- | --- | --- | --- | --- | --- | --- | --- | --- | --- |
| **Food groups** | **Total** | **Dietary Patterns** | | | | | | | | | | |
|  |  | **‘Western’**  **tertiles** | | | **‘Prudent’**  **tertiles** | | | **‘Processed plant fats and sweetened dairy’**  **tertiles** | | | **‘Polish-aMED’**  **levels** | |
|  |  | bottom | middle | upper | bottom | middle | upper | bottom | middle | upper | lower | higher |
| Sample size (n) | 420 | 139 | 141 | 140 | 139 | 140 | 141 | 140 | 139 | 141 | 189 | 231 |
| Frequency of food consumption (times/day)^#^ | | | | | | | | | | | | |
| Fish | 0.3  (0.2; 0.3) | 0.3  (0.2; 0.4) | 0.3  (0.2; 0.3) | 0.3  (0.2; 0.3) | 0.1  (0.1; 0.2) | 0.2  (0.2; 0.3) | 0.5  (0.4; 0.5) | 0.2  (0.2; 0.3) | 0.3  (0.2; 0.3) | 0.3  (0.2; 0.3) | 0.2  (0.2, 0.2) | 0.3  (0.3, 0.4) |
| Nuts and seeds | 0.3  (0.3; 0.4) | 0.5  (0.4; 0.6) | 0.3  (0.2; 0.3) | 0.2  (0.1; 0.2) | 0.1  (0.1; 0.2) | 0.3  (0.2; 0.4) | 0.6  (0.5; 0.7) | 0.4  (0.3; 0.5) | 0.4  (0.3; 0.5) | 0.3  (0.2; 0.3) | 0.1  (0.1, 0.1) | 0.5  (0.4, 0.6) |
| Legumes | 0.2  (0.2; 0.2) | 0.2  (0.2; 0.3) | 0.2  (0.2; 0.2) | 0.2  (0.2; 0.3) | 0.1  (0.1; 0.1) | 0.2  (0.1; 0.2) | 0.4  (0.3; 0.4) | 0.2  (0.1; 0.2) | 0.2  (0.2; 0.3) | 0.3  (0.2; 0.3) | 0.1  (0.1, 0.1) | 0.3  (0.3, 0.3) |
| Breakfast cereals | 0.2  (0.1; 0.2) | 0.1  (0.1; 0.2) | 0.2  (0.1; 0.2) | 0.2  (0.1; 0.2) | 0.0  (0.0; 0.1) | 0.2  (0.1; 0.2) | 0.3  (0.2; 0.3) | 0.1  (0.0; 0.1) | 0.2  (0.1; 0.2) | 0.3  (0.2; 0.3) | 0.1  (0.1, 0.2) | 0.2  (0.2, 0.2) |
| Sweetened beverages and energy drinks | 0.1  (0.0; 0.1) | 0.0  (0.0; 0.0) | 0.0  (0.0; 0.0) | 0.1  (0.1; 0.2) | 0.0  (0.0; 0.1) | 0.0  (0.0; 0.1) | 0.1  (0.1; 0.1) | 0.0  (0.0; 0.1) | 0.0  (0.0; 0.1) | 0.1  (0.1; 0.1) | 0.0  (0.0, 0.1) | 0.1  (0.0, 0.1) |

Polish-aMED – Polish-adapted Mediterranean Diet (range of points: 0-8), levels (in points): ‘lower’ (0-4), ‘higher’ (5-8); ^#^the frequency consumption was expressed as a times/day after assigning the values for categories of frequency consumption as follows: ‘never or almost never’=0; ‘once a month or less’=0.025; ‘several times a month’=0.1; ‘several times a week’=0.571; ‘daily’=1; ‘several times a day’=2; 95%CI – 95% confidence interval.

**Table S6. The mean (95% CI) of the consumption of food groups and the ratio of vegetable oils to animal fat by the level of nutritional knowledge among peri- and post-menopausal women (n=417), mean (SD)**

| Frequency of food consumption (times/day)^#^ | **Nutritional knowledge level (points)** | | | ***p*-Value** |
| --- | --- | --- | --- | --- |
|  | **low**  **(≤12)** | **average (13-15)** | **high**  **(≥16)** |  |
| Sample Size (n) | 142 | 141 | 134 |  |
| Refined cereals | 1.0 (0.9; 1.2) | 1.0 (0.9; 1.1) | 0.8 (0.7; 0.9) | 0.0092 |
| Red and processed meats | 1.5 (1.3; 1.6) | 1.4 (1.2; 1.5) | 1.2 (1.1; 1.4) | 0.0257 |
| Sugar, honey and sweets | 2.0 (1.8; 2.2) | 2.0 (1.7; 2.2) | 1.7 (1.5; 1.9) | 0.0820 |
| Potatoes | 0.7 (0.6; 0.7) | 0.6 (0.6; 0.7) | 0.5 (0.5; 0.6) | 0.0022 |
| Animal fats | 1.2 (1.0; 1.3) | 1.1 (1.0; 1.3) | 1.2 (1.0; 1.3) | 0.8222 |
| Vegetable oils (including olive oil) | 0.6 (0.5; 0.7) | 0.6 (0.6; 0.7) | 0.6 (0.6; 0.7) | 0.8271 |
| Sweetened beverages and energy drinks | 0.1 (0.0; 0.1) | 0.1 (0.0; 0.1) | 0.0 (0.0; 0.1) | 0.2642 |
| Fruit | 0.9 (0.8; 1.0) | 1.0 (0.9; 1.1) | 1.0 (0.9; 1.1) | 0.2751 |
| Fish | 0.3 (0.2; 0.3) | 0.3 (0.2; 0.3) | 0.3 (0.2; 0.4) | 0.3565 |
| Legumes | 0.2 (0.1; 0.2) | 0.2 (0.2; 0.3) | 0.2 (0.2; 0.3) | 0.0512 |
| Milk, fermented milk drinks and cheese curd | 0.9 (0.8; 1.1) | 1.2 (1.1; 1.3) | 1.1 (1.0; 1.2) | 0.0019 |
| Wholemeal cereals | 0.8 (0.7; 0.9) | 0.9 (0.7; 1.0) | 1.0 (0.9; 1.2) | 0.0107 |
| Fruit, vegetable, vegetable-fruit juices | 0.4 (0.3; 0.5) | 0.5 (0.4; 0.6) | 0.5 (0.4; 0.6) | 0.2678 |
| Eggs | 0.3 (0.3; 0.4) | 0.4 (0.3; 0.4) | 0.4 (0.3; 0.4) | 0.5014 |
| Vegetables | 1.1 (1.0; 1.2) | 1.2 (1.1; 1.3) | 1.4 (1.3; 1.5) | 0.0001 |
| Nuts and seeds | 0.3 (0.2; 0.3) | 0.3 (0.3; 0.4) | 0.4 (0.3; 0.5) | 0.0002 |
| Breakfast cereals | 0.2 (0.1; 0.2) | 0.2 (0.1; 0.2) | 0.1 (0.1; 0.2) | 0.3747 |
| Cheese | 0.4 (0.3; 0.4) | 0.4 (0.3; 0.4) | 0.4 (0.3; 0.4) | 0.5756 |
| Other fats (margarine, mayonnaise, dressings) | 0.6 (0.4; 0.7) | 0.5 (0.4; 0.6) | 0.3 (0.2; 0.4) | 0.0354 |
| Sweetened milk beverages and flavoured cheese | 0.3 (0.2; 0.4) | 0.4 (0.3; 0.5) | 0.2 (0.2; 0.3) | 0.0399 |
| White meat | 0.4 (0.4; 0.5) | 0.5 (0.4; 0.5) | 0.4 (0.4; 0.5) | 0.7525 |
| Ratio of vegetable oils to animal fat | 1.6 (1.0; 2.3) | 1.6 (0.9; 2.3) | 1.8 (1.0; 2.6) | 0.7249 |

^#^the frequency consumption was expressed as a times/day after assigning the values for categories of frequency consumption as follows: ‘never or almost never’=0; ‘once a month or less’=0.025; ‘several times a month’=0.1; ‘several times a week’=0.571; ‘daily’=1; ‘several times a day’=2; 95%CI – 95% confidence interval; p-value – level of significance assessed by the Kruskal-Wallis’ test.

**Table S7**. Description of food groups for the Polish-adapted Mediterranean Diet score (0-8 points) calculation – data for the Initial control sample [Krusinska et al. 2018]

| **Food groups/food items** | **Frequency of consumption** (times/day)* | | **Criteria for 1 point** |
| --- | --- | --- | --- |
|  | **Mean (95% CI)** | **Median** |  |
| **VEGETABLES**: all kinds of vegetables:  cruciferous vegetables (cabbages, brussel sprouts, cauliflower, broccoli, kale, etc.), yellow-orange vegetables (carrots, peppers, etc.), leafy green vegetables (spinach, chicory, lettuce, rocket, leek, celery, parsley, etc.), tomatoes, gourds and squashes (fresh cucumber, marrow, courgettes, pumpkins, aubergines, etc.), root vegetables and other (parsnip, beetroots, onion, garlic, celeriac, radishes, turnip, salads and mixed vegetables, etc.) | 1.300 (1.230; 1.371) | 1.000 | Greater than median intake (times/day)^*^ |
| **FRUIT**: all kinds of fruit:  stone fruit (apricots, cherries, nectarines, peaches, plums, grapes, etc.), kiwi and citrus fruit (kiwi, oranges, mandarins, grapefruit, lemons, pomelos, etc.), tropical fruits (pineapples, watermelon, melons, fresh dates and figs, etc.), berries (strawberries, raspberries, blackberries, blueberries, redcurrants, blackcurrants, etc.), bananas, apples, pears, avocado | 0.989 (0.925; 1.052) | 1.000 | Greater than median intake (times/day)^*^ |
| **WHOLEMEAL CEREALS**: wholemeal wheat or rye bread, seeded loafs, pumpernickel, wholemeal groats, wholemeal rice, wholemeal pasta | 0.890 (0.799; 0.981) | 0.671 | Greater than median intake (times/day)^*^ |
| **FISH**: lean fish (ollock, cod, perch, hake, carp to 1 kg, tuna, panga, trout, etc.)  oily fish (salmon, sardines, herring, mackerel, eel, large carp, etc.) | 0.293 (0.253; 0.334) | 0.200 | Greater than median intake (times/day)^*^ |
| **LEGUMES**: fresh and tinned legumes (corn, green peas, green beans, etc.),  dry and processed pulses beans (fava, broad, French, green), soya, peas, chickpea and processed pulses (baked beans, hummus, other bread spreads) | 0.223 (0.187; 0.259) | 0.125 | Greater than median intake (times/day)^*^ |
| **NUTS** and **SEEDS**: peanuts, hazelnuts, walnuts, almonds, pistachios, cashews, coconuts, chestnuts, etc., pumpkin seeds, sesame seeds, sunflower seeds, wheat germs, etc. | 0.375 (0.312; 0.438) | 0.125 | Greater than median intake (times/day)^*^ |
| **RATIO of VEGETABLES OILS** (rapeseed oil, sunflower oil, linseed oil, olives) **to ANIMAL FAT** (butter, cream, lard) instead of RATIO of MONOUNSATURATED to SATURATED FAT | 1.462 (1.125; 1.799) | 0.519 | Greater than median intake (times/day)^*^ |
| **RED** and **PROCESED MEAT**: red meat (pork, beef, veal, etc.), sausages, bacon, reconstituted meat (sausages, meat loaf, hot-dogs, smoked sausages, bacon, etc.), high quality cured meats (ham, poultry and pork-beef good quality cold meats, etc.), offal products (liver, blood sausage, sweetbread, liver pate, etc.), game (wild boar, venison, quail, mallard, hare, etc.) | 1.252 (1.151; 1.352) | 1.267 | Lower than median intake (times/day)^*^ |

*food frequency consumption was expressed as a times/day after assigning the values for categories of frequencies as follows: ‘never or almost never’=0; ‘once a month or less’=0.025; ‘several times a month’=0.1; ‘several times a week’=0.571; ‘daily’=1; ‘several times a day’=2; 95% CI – 95% confidence interval.

**Table S8**. Confounders in the case-control study regarding the association of nutrition knowledge, dietary patterns, and breast cancer occurrence among peri- and post-menopausal women [Krusinska et al. 2018]

| **Confounders** | **Categories** |
| --- | --- |
| **Age** (years) | 40.0-49.9; 50.0-59.9; 60.0-69.9; 70.0-79.9 |
| **BMI** (kg/m^2^)  calculated using measured weight and height | ≤24.9; 25.0-29.9; ≥30.0 |
| **Socioeconomic status (SES)**  after combining data based on SES’ factors: | low; average; high |
| place of residence | village |
|  | town <20,000 inhabitants |
|  | town 20,000-100,000 inhabitants |
|  | city >100,000 inhabitants |
| educational level | primary |
|  | secondary |
|  | higher |
| economic situation  (self-declared) | below average |
|  | average |
|  | above average |
| situation of household (self-declared) | we live poorly – I don’t have enough resources even for basic needs (food/clothing/housing fees) |
|  | we live very thriftily – I have enough resources only for basic needs (food/clothing/housing fees) |
|  | we live thriftily – so I have enough resources for everything |
|  | we live well – I have enough resources for everything, but I don’t put off savings |
|  | we live very well – I have enough resources for everything and I put off savings |
| **Menopausal status** | peri-, postmenopausal |
| **Age at menarche** (years) | <12.0; 12-14.9; ≥15.0 |
| **Oral contraceptive use** (ever) | no; yes |
| **Hormone-replacement therapy use** | no; yes |
| **Number of full-term pregnancies** | 0; 1-2; ≥3 |

| **Table S8.** *Cont.* | | |
| --- | --- | --- |
| **Overall physical activity**  after combining data based  on physical activity at work  and physical activity in leisure time | low; moderate; high | |
| physical activity at work | low | more than 70% of working time spent sedentary or retired |
|  | moderate | approx. 50% of working time spent sedentary and 50% of working time spent in an active manner |
|  | high | approx. 70% of working time spent in an active manner or physical work related to great exertion |
| physical activity in leisure time | low | sedentary for most of the time, watching TV, reading books, walking 1-2 hours per week |
|  | moderate | walking, bike riding, gymnastics, gardening, light physical activity performed 2-3 hours per week |
|  | high | bike riding, jogging, gardening, sport activities involving physical exertion performed more than 3 hours weekly |
| **Abuse of alcohol** | no |  |
|  | yes | intake at least 1 bottle (0.5 liters) of beer or 2 glasses of wine (300ml) or 2 drinks (300ml) or 2 glasses of vodka (60ml) per day |
| **Smoking** **status** | non-smoker |  |
|  | smoker | current-smoker or/ and former-smoker |
| **Vitamin/mineral supplements use**  (within last 12 months) | no; yes | |
| **Family history of breast cancer in first-**  **or second-degree relatives** | no; I don’t know; yes | |
| **Molecular of breast cancer subtypes** | triple negative; ER-, PR-, HER2+ subtype; luminal A; luminal B | |
